# Integrated Gradient Cu Current Collector Enables Bottom‐Up Li Growth for Li Metal Anodes: Role of Interfacial Structure

**DOI:** 10.1002/advs.202301288

**Published:** 2023-06-13

**Authors:** Yuhang Liu, Yifan Li, Zhuzhu Du, Chen He, Jingxuan Bi, Siyu Li, Wanqing Guan, Hongfang Du, Wei Ai

**Affiliations:** ^1^ Frontiers Science Center for Flexible Electronics and Shaanxi Institute of Flexible Electronics Northwestern Polytechnical University 127 West Youyi Road Xi'an 710072 China; ^2^ Fujian Cross Strait Institute of Flexible Electronics (Future Technologies) Fujian Normal University Fuzhou 350117 China

**Keywords:** bottom‐up deposition, Cu current collectors, gradient design, interfacial structure, Li metal batteries

## Abstract

3D Cu current collectors have been demonstrated to improve the cycling stability of Li metal anodes, however, the role of their interfacial structure for Li deposition pattern has not been investigated thoroughly. Herein, a series of 3D integrated gradient Cu‐based current collectors are fabricated by the electrochemical growth of CuO nanowire arrays on Cu foil (CuO@Cu), where their interfacial structures can be readily controlled by modulating the dispersities of the nanowire arrays. It is found that the interfacial structures constructed by sparse and dense dispersion of CuO nanowire arrays are both disadvantageous for the nucleation and deposition of Li metal, consequently fast dendrite growth. In contrast, a uniform and appropriate dispersity of CuO nanowire arrays enables stable bottom Li nucleation associated with smooth lateral deposition, affording the ideal bottom‐up Li growth pattern. The optimized CuO@Cu‐Li electrodes exhibit a highly reversible Li cycling including a coulombic efficiency of up to ≈99% after 150 cycles and a long‐term lifespan of over 1200 h. When coupling with LiFePO_4_ cathode, the coin and pouch full‐cells deliver outstanding cycling stability and rate capability. This work provides a new insight to design the gradient Cu current collectors toward high‐performance Li metal anodes.

## Graphical abstract

3D integrated gradient CuO@Cu nanowire arrays with varying dispersities are synthesized to investigate the correlation between interfacial structure and Li deposition pattern. It is found that the consistent bottom‐up Li deposition pattern is not only related to the gradient structure that is capable of bottom nucleation, but also requires an ideal interface for facilitating smooth lateral Li deposition.

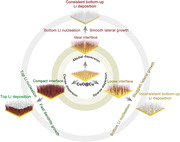

## 1 Introduction

Li metal anodes (LMAs) have sparked tremendous attention over recent decades in the community of high‐energy batteries because of the high theoretical capacity (3860 mAh g^−1^) and low redox potential (−3.04 V versus standard hydrogen electrode).^[^ ^1^ ^]^ Unfortunately, serious instability stemming from the huge electrode volume swings and parasitic dendrite growth poses fast capacity fading and safety hazards.^[^ ^2^ ^]^ Efforts toward stabilizing LMAs so far have been focused on hosting Li within 3D current collectors, which not only alleviate electrode swells but also homogenize Li‐ion flux for regulating the nucleation and deposition of Li.^[^ ^3^ ^]^ However, due to “tip effect”, visible dendrites remain observed in their inner parts, especially under high‐rate and high‐capacity conditions, rendering the practical deployment of LMAs untenable.^[^ ^4^ ^]^ It is well recognized that Li deposition pattern plays a vital role in determining the cycling stability and safety of the batteries.^[^ ^5^ ^]^ In an ideal deposition pattern, Li nucleation and growth follow a bottom‐up model, which could avoid the invalidation of 3D current collectors and the short‐circuit risk effectively.^[^ ^6^ ^]^ Given that Li deposition is a diffusion‐controlled reaction process dominated by the ion and electron fields, modulating the lithiophilicity and/or conductivity of 3D current collectors have been demonstrated to be viable routes to regulate the associated deposition patterns.^[^ ^7^ ^]^

Thus far, a variety of gradient current collectors designed with oriented conductivity and/or lithiophilicity have been explored to guide Li deposition.^[^ ^8^ ^]^ Among, 3D gradient Cu current collectors show great potential to speed up the practical use of LMAs in view of their facile fabrication and excellent conductivity.^[^ ^9^ ^]^ In this respect, insulating and lithiophilic networks have been used as the functional layers to cover Cu substrates, so that to selectively reinforce the electron and/or ion accumulations at the bottom of the current collectors for bottom‐up Li deposition.^[^ ^10^ ^]^ Despite substantial progress, several issues remain to be addressed. On one hand, the gradient Cu‐based current collectors are normally constructed by multilayered structures, which generate additional interfaces with increased interfacial resistances.^[^ ^11^ ^]^ On the other hand, the interfacial structures could be easily destroyed during long‐term cycling, resulting in uncontrolled Li plating/stripping processes and thus fast dendrite growth and battery failure.^[^ ^12^ ^]^ To date, the investigations of 3D gradient Cu current collectors remain at a nascent stage, and there is no clarity on how their interfacial structures influence Li nucleation and deposition. Our recent work has indicated that the Li plating/stripping behaviors have strong dependency on the structure of 3D skeletons.^[^ ^13^ ^]^ Therefore, it would be necessary and valuable to utilize integrated gradient Cu current collectors to study the correlation between interfacial structure and Li deposition pattern.

Herein, we developed 3D integrated architectures comprising CuO nanowire arrays with varying dispersities grown on Cu current collector (denoted as CuO@Cu) by means of a facile anodization and subsequent calcination, where the lithiophilic but semiconducting CuO nanowire arrays afford a gradient electron density distribution on the vertical direction of the scaffolds. Meanwhile, the dispersion differences of CuO nanowire arrays result in different interfacial structures that are significantly correlated with the Li deposition pattern. In terms of a bottom‐up pattern, Cu current collector with gradient structure that is capable of introducing bottom Li nucleation is necessary. More importantly, a unique interfacial structure consisting of uniform and appropriate dispersity of CuO nanowire arrays holds the key for durable LMAs since it dominates the lateral growth of Li metal. The optimized CuO@Cu nanowire arrays show a low nucleation overpotential of ≈12 mV, a high coulombic efficiency (CE) of ≈99% after 150 cycles, and a long‐term cyclability for over 1200 h. This work may provide guidance for the rational design of gradient Cu current collectors with stabilized bottom‐up Li growth toward highly reversible LMAs.

## 2 Results and Discussion

**Figure 1:**
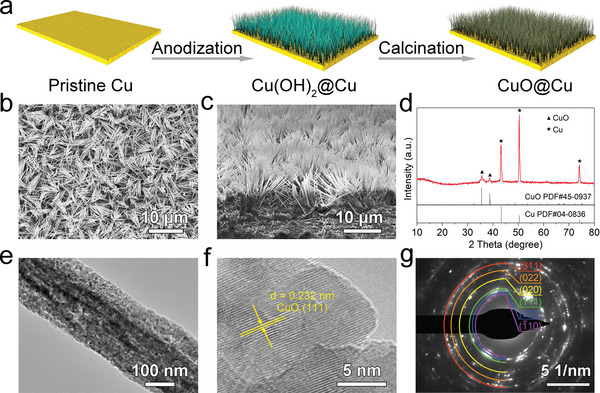
a) Schematic illustration of the preparation of CuO@Cu nanowire arrays. Scanning electron microscopy (SEM) images of the b) surface view and c) cross‐section view of M‐CuO@Cu nanowire arrays. d) X‐ray diffraction (XRD) pattern, e) transmission electron microscopy (TEM), f) high‐resolution TEM (HRTEM), and g) the corresponding selected area electron diffraction (SAED) images of M‐CuO@Cu nanowire arrays.

The CuO@Cu nanowire arrays were fabricated by the anodization of Cu foils in 2 m KOH electrolyte, followed by calcination at 200 °C for 2 h (**Figure** 1a). During the anodization process, Cu foil was oxidized to release Cu^2+^ for promoting the growth of Cu(OH)_2_ nanowire arrays.^[^ ^14^ ^]^ The interfacial structure of the as‐grown scaffolds can be readily controlled by tunning the dispersities of nanowire arrays. As shown in the scanning electron microscopy (SEM) images in Figure S1a–c (Supporting Information), when anodizing at 6 mA cm^−2^ for 100 s, the Cu(OH)_2_ nanowire arrays are sparsely grown on the Cu current collectors (S‐Cu(OH)_2_@Cu). The large gap between neighboring nanowire arrays leaves plenty of exposed substrate, hence a loose interface. At a longer reaction time of 500 s, the increased nucleation density of Cu(OH)_2_ nanowire arrays produces medially dispersed Cu(OH)_2_ nanowire arrays with uniform coverage on Cu substrate (M‐Cu(OH)_2_@Cu) (Figure S1d–f, Supporting Information). Accordingly, the appropriate dispersity of nanowire arrays leads to a unique interface with uniform and consistent features. Further increasing the reaction time to 1000 s, Cu(OH)_2_ nanowire arrays with more densely dispersed structure are noted (D‐Cu(OH)_2_@Cu), which gives rise to a compact interface due to its narrow arrays spacing (Figure S1g–i, Supporting Information). After calcination, the resultant CuO nanowire arrays well inherit the structures from Cu(OH)_2_ precursors nanowire arrays (Figure 1b; Figure S2, Supporting Information). The as‐obtained samples were denoted as S‐CuO@Cu, M‐CuO@Cu, and D‐CuO@Cu, according to the interfacial structure of CuO nanowire arrays. The cross‐section SEM images present that the CuO nanowire arrays show good integration with the Cu substrate, which ensures good electrical contact and structural stability (Figure 1c and Figure S3, Supporting Information). As depicted in Figure S4 (Supporting Information), all the integrated CuO@Cu nanowire arrays deliver higher surface resistances than bare Cu substrate, rendering a vertical conductivity gradient that is beneficial to the bottom‐up Li deposition. In the following part, M‐CuO@Cu is used as the main example to describe the characterizations. X‐ray diffraction (XRD) pattern in Figure 1d exhibits two diffraction peaks at 35.5° and 38.7°, which are well index to the (002) and (111) planes of CuO (PDF#45‐0937). Figure 1e shows the transmission electron microscopy (TEM) image of a typical nanowire of the arrays, where a porous feature is observed. Such an unusual structure would be beneficial for the exposure of active sites, contributing to the outstanding electrochemical performances of M‐CuO@Cu. High‐resolution TEM (HRTEM) image demonstrates a lattice fringe of 0.232 nm, corresponding to the (111) plane of CuO (Figure 1f). Furthermore, the selected area electron diffraction (SAED) pattern also well coincides with the CuO phase, consistent with the XRD results (Figure 1g). These results imply the successful synthesis of CuO nanowire arrays.

To comprehensively evaluate the lithiophilicity of CuO@Cu nanowire arrays, the Li ions migration behaviors and wettability with the liquefied Li metal were performed. First, electrochemical surface area (ECSA) was served as a quantitative indicator to analyze the density of lithiophilic sites based on the double‐layer capacitances (*C*_dl_).^[^ ^15^ ^]^ Figure S5 (Supporting Information) shows the estimated *C*_dl_ values are 9.19 mF cm^−2^ for S‐CuO@Cu, 23.75 mF cm^−2^ for M‐CuO@Cu and 43.20 mF cm^−2^ for D‐CuO@Cu, which demonstrates that the normalized ECSA increases with the increasing of arrays density. The increase of ECSA guarantees more exposure of active sites per unit area, consequently a higher Li affinity.^[^ ^16^ ^]^ As revealed by molten Li infusion tests, M‐CuO@Cu and D‐CuO@Cu could be completely covered by liquefied Li with a time of 70 and 35 s, respectively (Figure S6, Supporting Information). However, S‐CuO@Cu only delivers a short‐range infusion even with a longer time of 160 s. Note that, bare Cu is very difficult to be wetted by liquefied Li in view of its lithiophobic nature. Contact angle measurements were further conducted to evaluate the influence of interfacial structure on the electrolyte wettability. Figure S7 (Supporting Information) presents that the contact angles of bare Cu, S‐CuO@Cu, M‐CuO@Cu, and D‐CuO@Cu are determined to be ≈90°, ≈26°, ≈10°, and ≈0°, respectively. The significantly smaller value of CuO@Cu nanowire arrays than bare Cu suggests their good electrolyte wettability, which is supposed to improve the rate performance of the electrodes. In particular, the increase of arrays density contributes to a tighter interfacial structure and thus a better electrolyte wettability, agreeing well with the ECSA and molten Li infusion results.

**Figure 2:**
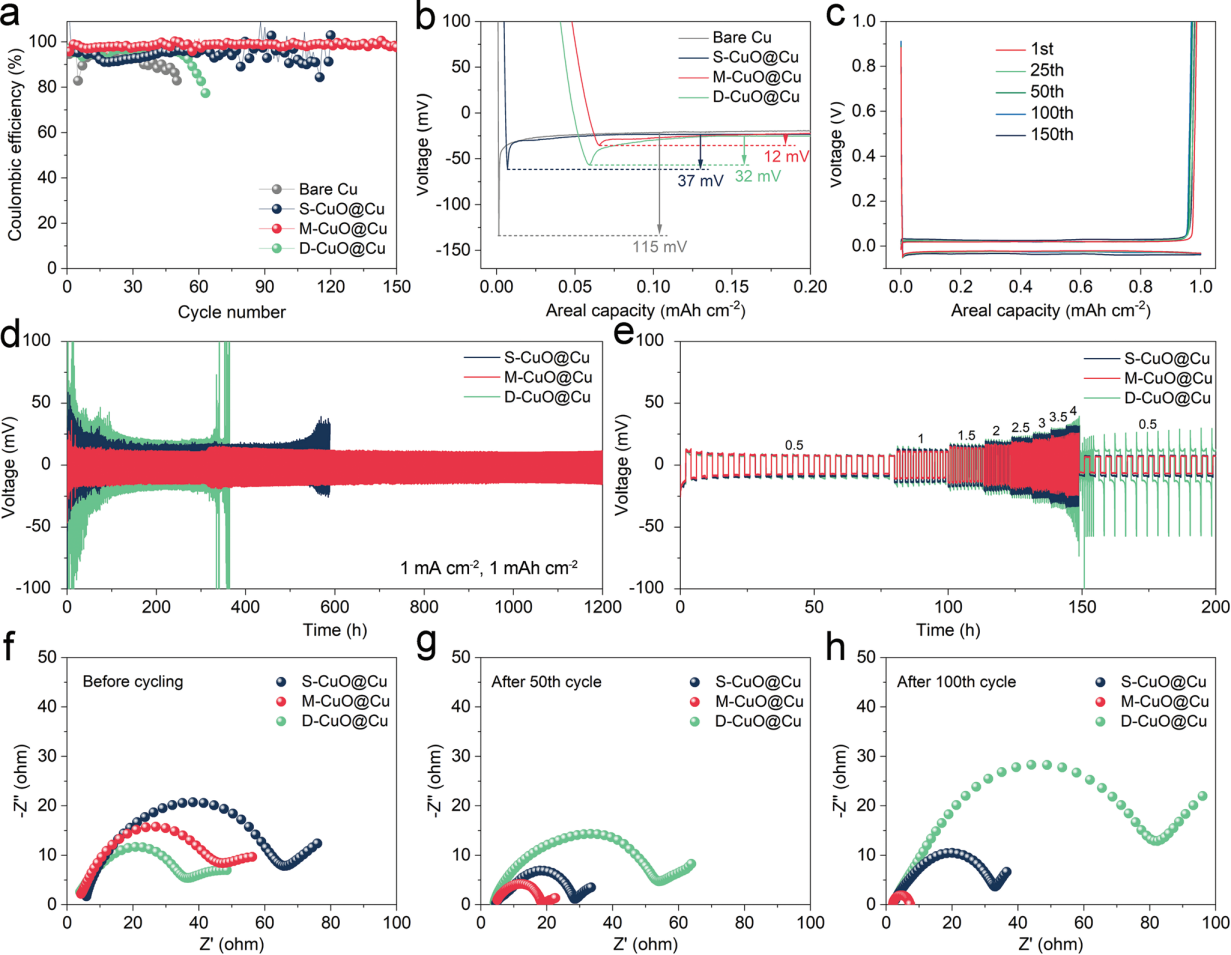
Comparison of the a) coulombic efficiency (CE) and b) nucleation overpotential for different electrodes at 1 mA cm^−2^ and 1 mAh cm^−2^. c) The corresponding voltage curves of M‐CuO@Cu electrode. d) Cycling and e) rate performance of the symmetric cells based on CuO@Cu nanowire arrays electrodes. Nyquist plots of the symmetric cells f) before cycling, and after g) 50th and h) 100th cycles at 1 mA cm^−2^ and 1 mAh cm^−2^.

**Figure** **2**a shows the CE profiles of different electrodes at a current density of 1 mA cm^−2^ and an area capacity of 1 mAh cm^−2^. Owing to the intrinsic lithiophobicity, bare Cu current collector offers an inferior CE below 90% and fast battery failure within 50 cycles. Meanwhile, the nucleation overpotential reaches as high as 115 mV (Figure 2b). In contrast, the CuO@Cu nanowire arrays showing different interfacial structures afford 3D confined space and plentiful lithiophilic sites, which lower the nucleation overpotential and improve the CE drastically. Specifically, M‐CuO@Cu electrode presents a stable and the highest CE of ≈99% during 150 cycles associated with the lowest nucleation overpotential of only 12 mV. While the S‐CuO@Cu delivers a high nucleation overpotential of 37 mV and a rapid decline of CE after 80 cycles. It is worth mentioning that D‐CuO@Cu holding the best lithiophilicity and electrolyte wettability exhibits an obvious fluctuation of CE and a moderate nucleation overpotential of 32 mV, which are the results of its compact interface that blocks Li nucleation within current collectors. The charge/discharge curves of M‐CuO@Cu electrode in Figure 2c appear to be almost overlapped within 150 cycles, which reflect its highly reversible Li plating/stripping processes. Even at a higher current density (3 mA cm^−2^) or area capacity (3 mAh cm^−2^), M‐CuO@Cu still maintains a steady CE and cycling performance (Figure S8, Supporting Information). This is mainly associated with the stable bottom‐up Li deposition pattern modulated by its decent interfacial structure (see the detailed discussions below).

The cycling stability of CuO@Cu‐Li electrodes was further investigated based on symmetric cells. Figure 2d compares the voltage profiles of different electrodes at a current density of 1 mA cm^−2^ with a capacity of 1 mAh cm^−2^. It is noted that M‐CuO@Cu‐Li displays superior cycling performance with a low overpotential of ≈10 mV for over 1200 h. Such a merit is more pronounced under a higher current density of 2 mA cm^−2^, where it maintains a low overpotential of 24 mV for over 500 h (Figure S9, Supporting Information). As summarized in Table S1 (Supporting Information), the cycling stability of M‐CuO@Cu‐Li electrode significantly surpasses previously reported functionalized Cu current collectors. By comparison, the S‐CuO@Cu‐Li electrode exhibits poor cyclic lifespan with a quick increase of hysteresis voltage and ultimately fails within 500 h at 1 mA cm^−2^ and 100 h at 2 mA cm^−2^. This can be attributed to the increased interfacial resistance caused by electrical disconnection and electrolyte depletion because of Li dendrites and/or dead Li accumulation.^[^ ^17^ ^]^ Worse still, D‐CuO@Cu‐Li shows the dramatic curve fluctuations and sudden voltage drop in the initial cycle stage at the current densities of 1 and 2 mA cm^−2^, which result from the fast Li dendrite growth and internal short circuit.^[^ ^18^ ^]^ To gain a deeper insight into the structural stability, the morphological evaluation of electrodes was further analyzed after 200 cycles at 1 mA cm^−2^ and 1 mAh cm^−2^. As shown the cross‐section SEM images in Figure S10 (Supporting Information), both S‐CuO@Cu‐Li and D‐CuO@Cu‐Li electrodes exhibit numerous Li dendrites and dead Li accumulation, while M‐CuO@Cu‐Li sustains its integrated nanowire arrays without any Li dendrite generation. Further rate tests of the electrodes were performed at the current densities ranging from 0.5 to 4 mA cm^−2^ with a fixed capacity of 1 mAh cm^−2^. As displayed in Figure 2e, the S‐CuO@Cu‐Li and D‐CuO@Cu‐Li electrodes suffer rigorous polarization, especially at a high current density of 4 mA cm^−2^. While the M‐CuO@Cu‐Li keeps a square‐wave‐shaped profile with a consistently low hysteresis voltage. The reposeful voltage polarization reflects its fast Li‐ion transport and smooth plating/stripping processes. In addition, the interfacial reaction kinetics were detected by electrochemical impedance spectroscopy (EIS) measurements, and the simulated equivalent circuit is shown in Figure S11 (Supporting Information). The semicircle in the high‐frequency region is related to the charge transfer resistance (*R*_ct_).^[^ ^19^ ^]^ Before cycling, the Nyquist plots manifest that the *R*_ct_ gradually decreases with the increase of CuO nanowire arrays density (Figure 2f and Figure S12, Supporting Information). This situation is because its compact interface provides more active sites for facilitating the charge transfer and Li‐ion diffusion kinetics. However, after 50 and 100 cycles, M‐CuO@Cu delivers lower *R*_ct_ as compared to the S‐CuO@Cu and D‐CuO@Cu electrodes, which represent its fast interfacial reaction kinetics that is associated with flat Li plating/stripping processes (Figure 2g,h). In contrast, S‐CuO@Cu delivers a decreased *R*_ct_ after 50 cycles but then an increased *R*_ct_ after 100 cycles, while D‐CuO@Cu sustains a constantly increased trend, both of which might provoke the accumulation of dendrites and dead Li.^[^ ^20^ ^]^

**Figure 3:**
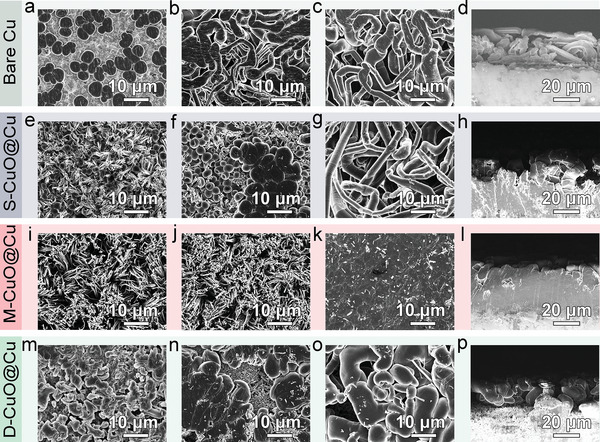
Scanning electron microscopy (SEM) images of a–d) bare Cu, e–h) S‐CuO@Cu, i–l) M‐CuO@Cu, and m–p) D‐CuO@Cu after Li plating: a,e,i,m) 0.25 mAh cm^−2^, b,f,j,n) 1 mAh cm^−2^, c,g,k,o) 3 mAh cm^−2^, and d,h,l,p) the corresponding cross‐section views.

To clarify the reason for mismatch between the lithiophilicity and electrochemical properties, the morphological evolution of CuO@Cu nanowire arrays during Li plating/stripping was explored at a current density of 0.25 mA cm^−2^. At a plating capacity of 0.25 mAh cm^−2^, numerous discrete Li nucleuses are randomly grown on bare Cu current collector surface (**Figure** **3**a). Since Li nucleation strongly affects the subsequent deposition, these uneven Li nucleuses quickly propagate into massive whisker‐like Li dendrites (Figure 3b–d).^[^ ^13^, ^21^ ^]^ In the case of CuO nanowire arrays, S‐CuO@Cu shows uniform Li nucleation at the bottom of the 3D nanowire arrays due to its conductivity gradient structure (Figure 3e). However, after 1 mAh cm^−2^ Li plating, Li clusters commence growing upwards from the individual isolated nanowires (Figure 3f), consequently resulting in severe dendrites accumulation under a large capacity of 3 mAh cm^−2^ (Figure 3g,h). Intriguingly, M‐CuO@Cu exhibits stable and uniform bottom nucleation of Li metal, accompanied with homogenous SEI layer formation (Figure 3i and Figure S13, Supporting Information). Even after 1 mAh cm^−2^ Li plating, it only depicts slight morphology changes (Figure 3j), which is mainly ascribed to the uniform lateral Li deposition at the bottom of the nanowire arrays. At a deposition capacity of 3 mAh cm^−2^, Li metal gradually deposits to fill up the inner space of the CuO nanowire arrays following a constant bottom‐up pattern, affording a flat and compact Li deposition morphology (Figure 3k,l). Regarding D‐CuO@Cu nanowire arrays, although it exhibits superior lithiophilicity, the nucleation of Li metal toward the bottom of current collectors was impeded by its compact interface. Therefore, a distinct top‐nucleation behavior with random Li nucleuses distribution was observed on the surface of the nanowire arrays (Figure 3m). These Li nucleuses acting as the “hot spots” trigger continual accumulation of Li metal (Figure 3n), and finally erupts the rapid growth of Li dendrites (Figure 3o,p). To better reveal the Li plating/stripping behaviors in these electrodes, their morphologies after fully Li stripping were subsequently studied (Figure S14, Supporting Information). Compared with the clear dead Li residues in the counterpart electrodes, the overall structure of M‐CuO@Cu could be recovered, indicative of its excellent structural stability and Li cycling reversibility. This could be responsible for the exceptional electrochemical performance of the M‐CuO@Cu electrode.

**Figure 4:**
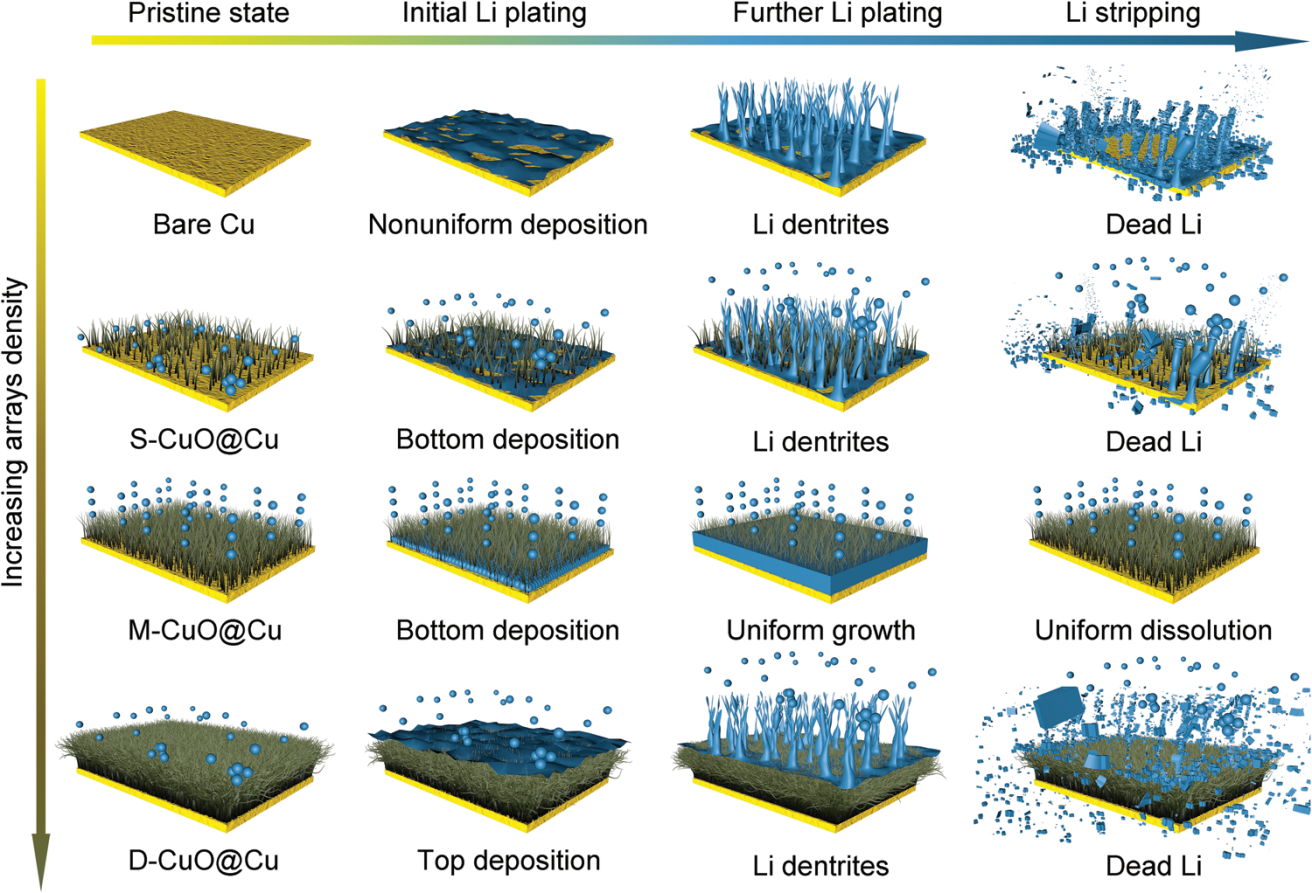
Schematic illustration of the Li deposition patterns on bare Cu, S‐CuO@Cu, M‐CuO@Cu, and D‐CuO@Cu electrodes.

**Figure** **4** shows the schematic illustration of Li plating/stripping processes within the bare Cu and CuO@Cu nanowire arrays electrodes. Due to the intrinsic lithiophobicity and rough surface, bare Cu current collector generates heterogeneous Li nucleation and hence rampant dendrite growth.^[^ ^22^ ^]^ While for the CuO@Cu nanowire arrays, the 3D integrated gradient structure could selectively reinforce charge distribution at the bottom of electrodes, causing oriented Li deposition in theory.^[^ ^9b^ ^]^ Note that, the interfacial structure constructed by CuO nanowire arrays also plays a vital role in determining the Li deposition pattern. For S‐CuO@Cu with sparse dispersion of the nanowire arrays, the resulting interface can readily achieve initial bottom nucleation of Li metal. Nonetheless, the large gap between neighboring nanowire arrays causes more exposure of lithiophobic interfacial region that is against the radial diffusion of Li ions, resulting in isolated Li clusters and subsequent dendrite formation. In turn, the top channels in D‐CuO@Cu are blocked during Li nucleation because of its compact interface, which inhibits Li deposition toward the bottom of the electrode, consequently triggering a top Li growth manner. Accordingly, both S‐CuO@Cu and D‐CuO@Cu electrodes display severely degraded electrochemical performance. Thanks to the uniform and appropriate arrays dispersity in M‐CuO@Cu, the associated ideal interface well promotes the stable bottom nucleation and then smooth lateral deposition of Li metal, which induces the anticipated bottom‐up Li deposition pattern with good reversibility.

**Figure 5:**
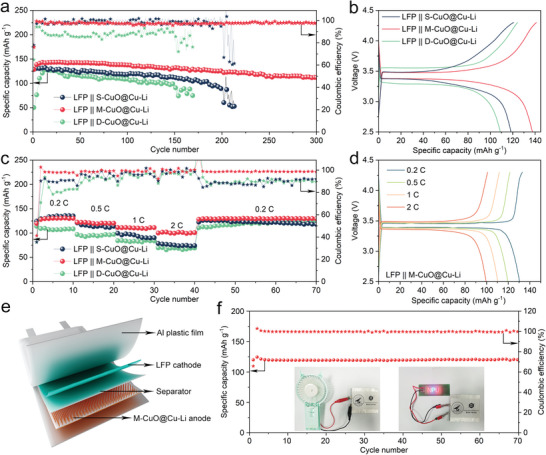
a) Cycling performance of LFP || CuO@Cu‐Li full‐cells at 1 C and b) the corresponding voltage curves after 100 cycles. c) Rate performance of LFP || CuO@Cu‐Li full‐cells and d) the corresponding voltage curves at different rates. e) Schematic of the LFP || M‐CuO@Cu‐Li pouch cell. f) Cycling performance of the LFP || M‐CuO@Cu‐Li pouch cell at 0.5 C. The insets are the digital photos of the pouch cell for powering electric fan and light‐emitting diodes (LEDs) lights.

To assess the prospect of CuO@Cu‐Li anodes for practical batteries, full‐cells were assembled by coupling with the LiFePO_4_ (LFP) cathode. As expected, the LFP || M‐CuO@Cu‐Li full‐cells show the best performance in terms of a high capacity of 112 mAh g^−1^ with a capacity retention of ≈88% after 300 cycles at 1 C (1 C = 170 mA g^−1^, **Figure** **5**a). In contrast, the LFP || S‐CuO@Cu‐Li and LFP || D‐CuO@Cu‐Li full‐cells deliver considerably lower capacities, with less than 90 mAh g^−1^ retained after 150 and 200 cycles, respectively. Figure 5b exhibits the charge/discharge curves of the full cells after 100 cycles. Compared with the other two counterparts, the smaller polarization voltage of LFP || M‐CuO@Cu‐Li indicates its faster electrochemical reaction kinetics, in agreement with the symmetric cell results. In addition, the full cells after 150 cycles at 1 C were disassembled to evaluate the structural stability of LFP cathode. The negligible morphology changes before and after cycling prove that the improvement of cycling performance mainly originates from the CuO@Cu‐Li anodes (Figure S15, Supporting Information). Further rate tests manifest that the LFP || M‐CuO@Cu‐Li full‐cells could release consistently higher discharge capacities of 130, 120, 110, and 100 mAh g^−1^ at 0.2, 0.5, 1, and 2 C, respectively (Figure 5c). After programmed rate tests, the discharge capacity of the LFP || M‐CuO@Cu‐Li can be completely restored when the rate goes back to 0.2 C. The corresponding voltage curves display slight polarization upon the increase of current rate, indicative of its outstanding cycling stability and rate capability (Figure 5d). Furthermore, LFP || M‐CuO@Cu‐Li pouch cells were assembled, which deliver an impressive capacity of 120 mAh g^−1^ and a capacity retention of up to 99.4% after 70 cycles at 0.5 C (Figure 5e,f). Importantly, the pouch cells can not only constantly drive a 3 V electric fan but also 30 parallel‐connected red light‐emitting diodes (LEDs) lights (inset of Figure 5f), indicating the potential applications of M‐CuO@Cu‐Li in practical Li metal batteries.

## 3 Conclusion

In summary, we designed a series of 3D integrated gradient CuO@Cu nanowire arrays with varying dispersities to clarify the correlation between interfacial structure and Li deposition pattern. It is demonstrated that the sparse dispersion of CuO nanowire arrays leads to a loose interface, which can induce bottom nucleation but impede lateral deposition of Li metal to provoke isolated Li clusters and dendrite formation. In turn, the densely dispersed CuO nanowire arrays will produce a compact interface that can pose the adverse top Li deposition pattern, resulting in fast dendrite growth. When the CuO@Cu achieves a uniform and appropriate dispersity, the resulting interface with uniform and consistent features could guarantee stable bottom nucleation and then smooth lateral deposition of Li metal, consequently an endurable bottom‐up Li deposition pattern. Accordingly, the optimized CuO@Cu nanowire arrays electrodes deliver a high CE of ≈99% after 150 cycles and stably cycle for more than 1200 h. Moreover, the full‐cells with LFP cathode achieve capacity retention of ≈88% after 300 cycles at 1 C. We believe this finding will provide new insights into the regulation of bottom‐up Li deposition pattern for high‐performance LMAs.

## 4 Experimental Section

### Preparation of Cuo@Cu Nanowire Arrays

The CuO@Cu nanowire arrays were prepared by anodization associated with a subsequent calcination. First, a 4 × 4 cm^2^ Cu foil (99.9%, 50 µm in thickness) was cleaned by consecutively sonicating in 1 m HCl solution, deionized water, and ethanol. A three‐electrode system was setup for the anodization, where the as‐obtained Cu foil was used as the working electrode. A stainless steel net, a saturated calomel electrode, and a 2 m KOH solution were employed as the counter electrode, reference electrode, and electrolyte, respectively. Cu(OH)_2_@Cu nanowire arrays were firstly prepared via chronopotentiometry under the current density of 6 mA cm^−2^ and programmed time. Subsequently, CuO@Cu nanowire arrays were obtained by the calcination of Cu(OH)_2_@Cu nanowire arrays at 200 °C for 2 h with a heating rate of 2 °C min^−1^ in an Ar flow of 100 sccm.

### Material Characterizations

SEM measurements were conducted using a field‐emission scanning electron microscope (FEI Verios G4) at an accelerating voltage of 10 kV. TEM analysis was performed on a transmission electron microscope (FEI Talos F200X) with an accelerating voltage of 200 kV. XRD patterns were measured on a Bruker D8 advance diffractometer with Cu K_*α*_ radiation (*λ* = 1.54056 Å). Contact angles tests were performed on a KRUSS DSA25 from 0–180°.

### Electrochemical Measurements

All cells were assembled in an Ar‐filled glove box (H_2_O < 0.1 ppm, O_2_ < 0.1 ppm) and tested on a NEWARE battery testing system. Celgard 2400 membrane was used as the separator. The electrolyte (80 µL for each cell) was 1 m lithium bis(trifluoromethanesulfonyl)imide in 1,3‐dioxolane and 1,2‐dimethoxyethane (1:1 in volume) with 1 wt% LiNO_3_. The half‐cells were assembled using CuO@Cu nanowire arrays as the working electrodes and Li foil as the counter electrode. Prior to cycling, all the cells were cleaned by precycling at 0.01–1.0 V and 0.05 mA cm^−2^ for five cycles. For CE tests, 1 mAh cm^−2^ of Li metal was deposited and then stripped until the cutoff voltage reaches 1.0 V. The ECSA was measured based on the cyclic voltammetry tests of half‐cells at different scan rates using a CHI 760E electrochemical workstation.

For symmetric cells assembly, two identical fresh Li foils or CuO@Cu‐Li electrodes with predeposited 3 mAh cm^−2^ of Li were used. The cycling performance tests were conducted based on the symmetric cells under programmed parameters. EIS was measured by an Autolab electrochemical workstation in the frequency range from 100 kHz to 0.01 Hz under open circuit potential. For full‐cells assembly, LFP was employed as the cathode (areal loading = 3 mg cm^−2^). The slurry was prepared by mixing LFP, super P, and polyvinylidene fluoride (8:1:1 in weight) in *N*‐methylpyrrolidone, and then coated on the Al foil before drying at 110 °C overnight under vacuum. The cycling and rate tests of full‐cells were operated at a potential range of 2.5–4.3 V.

## Conflict of Interest

The authors declare no conflict of interest.

## Supporting information

Supporting Information

## Data Availability

The data that support the findings of this study are available from the corresponding author upon reasonable request.
