# Supplementary material for: Integrated Gradient Cu Current Collector Enables Bottom‐Up Li Growth for Li Metal Anodes: Role of Interfacial Structure
Source: Adv Sci (Weinh). 2023 Jun 13;10(23):2301288. doi: 10.1002/advs.202301288 (PMC10427400; doi:10.1002/advs.202301288)
Supplement: Supplementary file 1 — Supporting Information [file ADVS-10-2301288-s001.pdf]

ADVANCED SCIENCE
Open Access

Supporting Information

for *Adv. Sci.*, DOI 10.1002/advs.202301288

Integrated Gradient Cu Current Collector Enables Bottom-Up Li Growth for Li Metal Anodes: Role of Interfacial Structure

Yuhang Liu, Yifan Li, Zhuzhu Du, Chen He, Jingxuan Bi, Siyu Li, Wanqing Guan, Hongfang Du and Wei Ai**

Supporting Information

Integrated gradient Cu current collector enables bottom-up Li growth for Li metal anodes: role of interfacial structure

Yuhang Liu, Yifan Li, Zhuzhu Du, Chen He, Jingxuan Bi, Siyu Li, Wanqing Guan, Hongfang Du and Wei Ai**

Mr. Y. Liu, Mr. Y. Li, Dr. Z. Du, Mr. C. He, Mr. J. Bi, Ms. S. Li, Ms. W. Guan, Prof. W. Ai
Frontiers Science Center for Flexible Electronics and Shaanxi Institute of Flexible Electronics, Northwestern Polytechnical University, 127 West Youyi Road, Xi'an 710072, China.
E-mail: iamzzdu@nwpu.edu.cn, iamwai@nwpu.edu.cn

Prof. H. Du
Fujian Cross Strait Institute of Flexible Electronics (Future Technologies), Fujian Normal University, Fuzhou 350117, China.

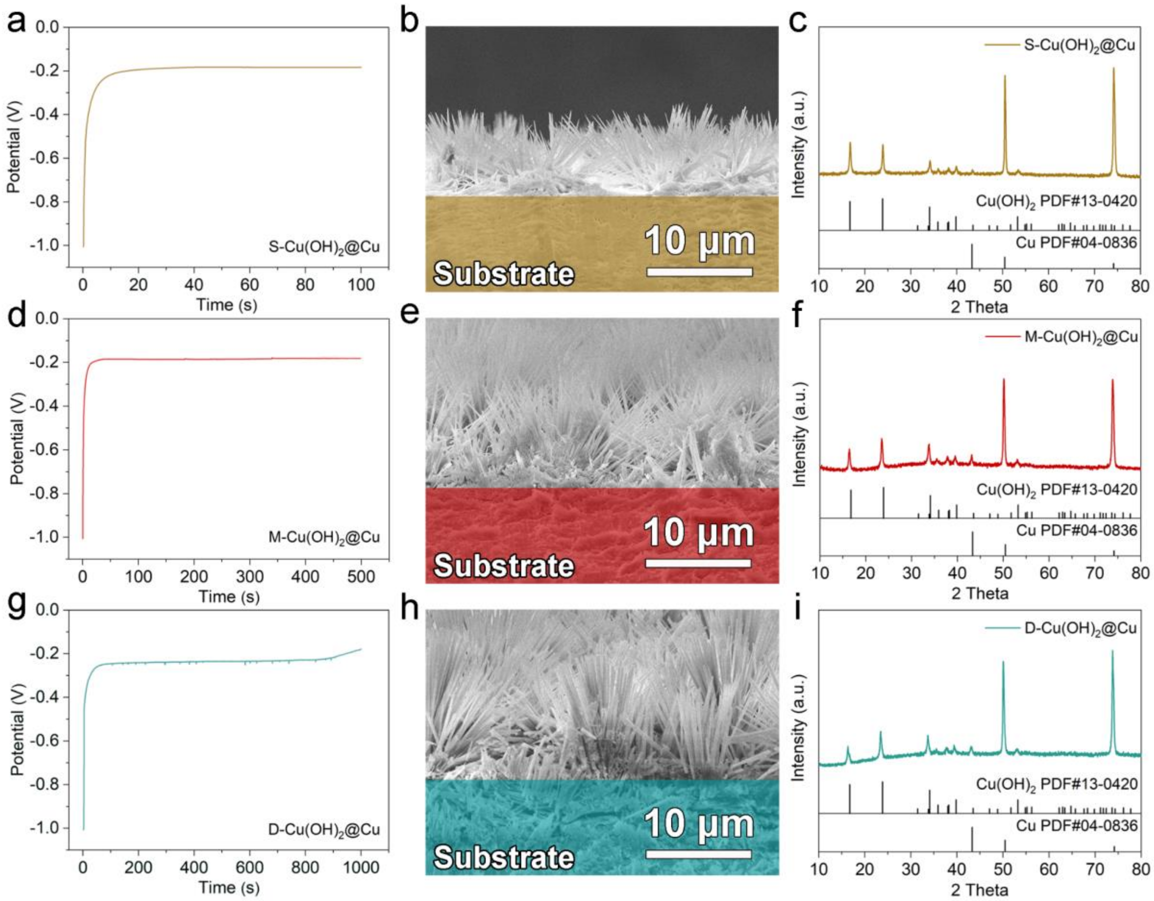

Figure S1. The preparation and characteristics of (a-c) S-$Cu(OH)_2$@Cu, (d-f) M-$Cu(OH)_2$@Cu and (g-i) D-$Cu(OH)_2$@Cu: (a, d, g) curves of galvanostatic anodization, (b, e, h) SEM images and (c, f, i) XRD patterns.

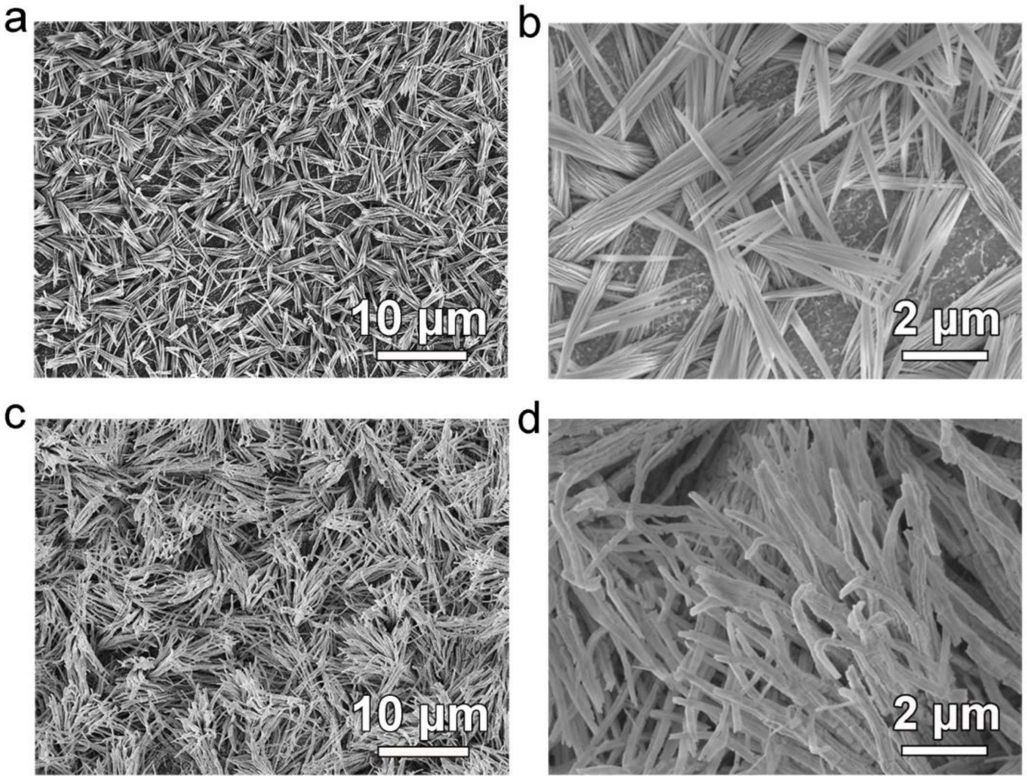

Figure S2. Surface SEM images of (a, b) S-CuO@Cu and (c, d) D-CuO@Cu nanowire arrays.

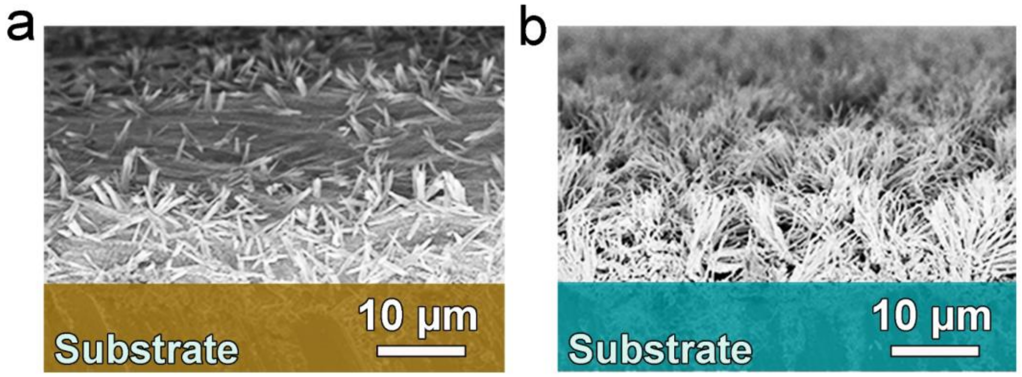

Figure S3. Cross-section SEM images of (a) S-CuO@Cu and (b) D-CuO@Cu nanowire arrays.

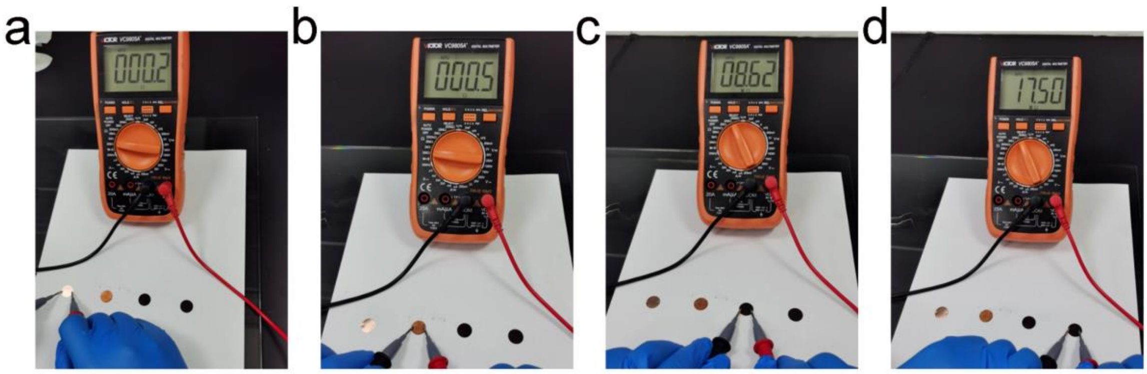

Figure S4. Digital photographs of resistance measures with digital multimeter for (a) bare Cu, (b) S-CuO@Cu, (c) M-CuO@Cu, (d) D-CuO@Cu nanowire arrays. All samples were punched into a circle with a diameter of 12 mm and the two test probes were maintained at a fixed length of 10 mm.

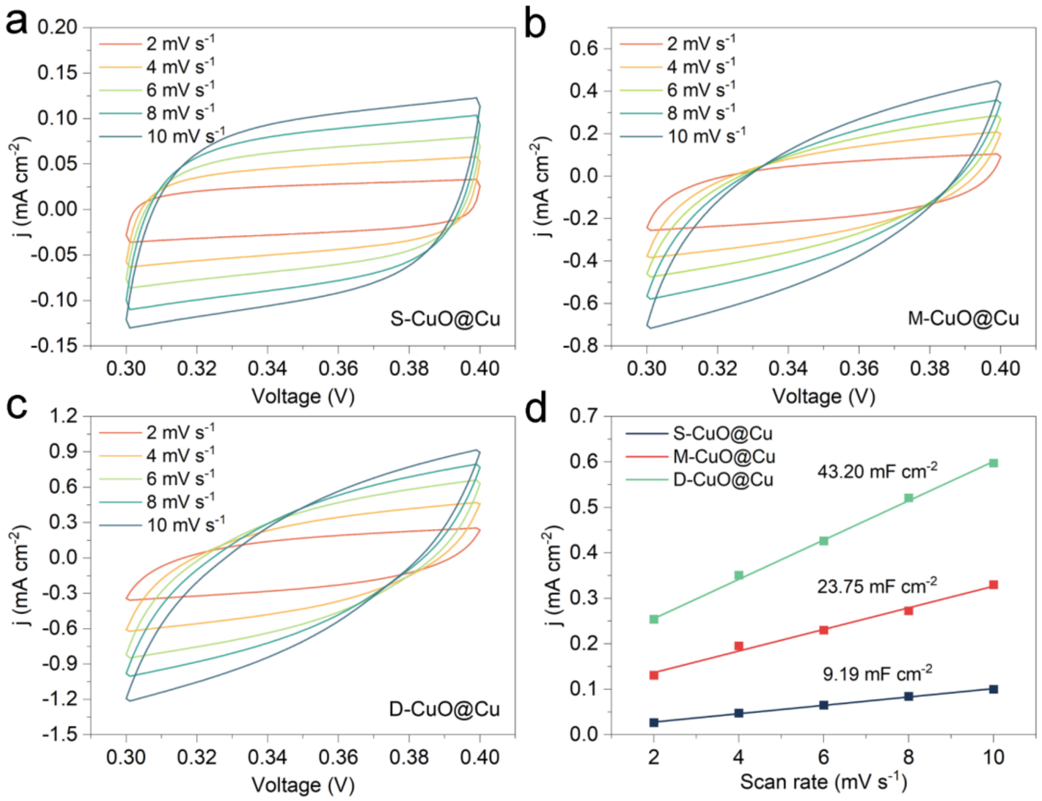

Figure S5. Cyclic voltammetry tests of half cells based on the (a) S-CuO@Cu, (b) M-CuO@Cu, (c) D-CuO@Cu electrodes at programmed scan rates. (d) The estimation of C_{dl} by plotting the current density variation against scan rate.

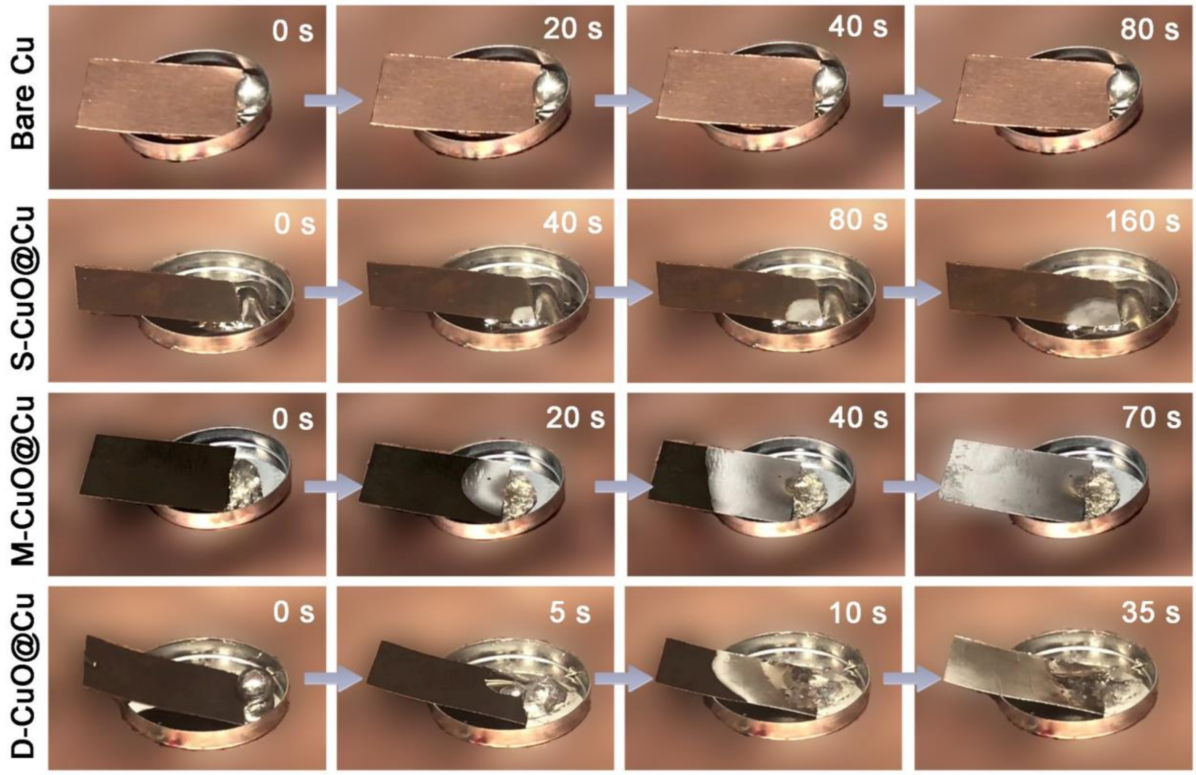

Figure S6. Digital photographs of molten Li infusion. All procedures were conducted on a hotplate with 300 °C in an argon-filled glove box.

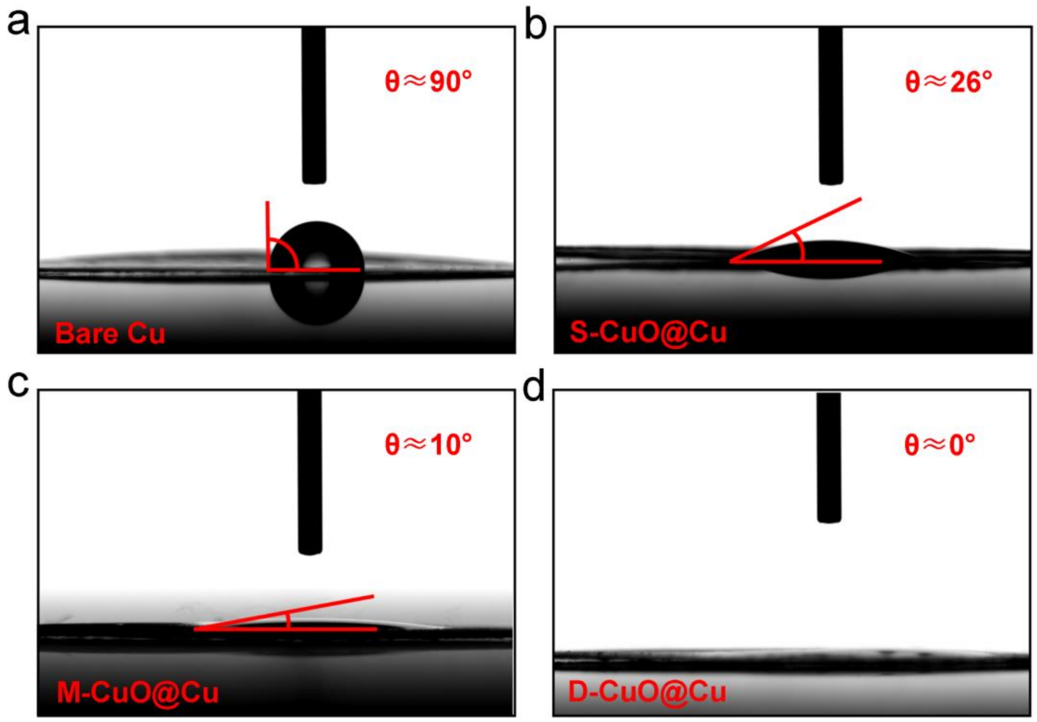

Figure S7. Contact angle tests of (a) bare Cu, (b) S-CuO@Cu, (c) M-CuO@Cu and (d) D-CuO@Cu nanowire arrays with the electrolyte solution.

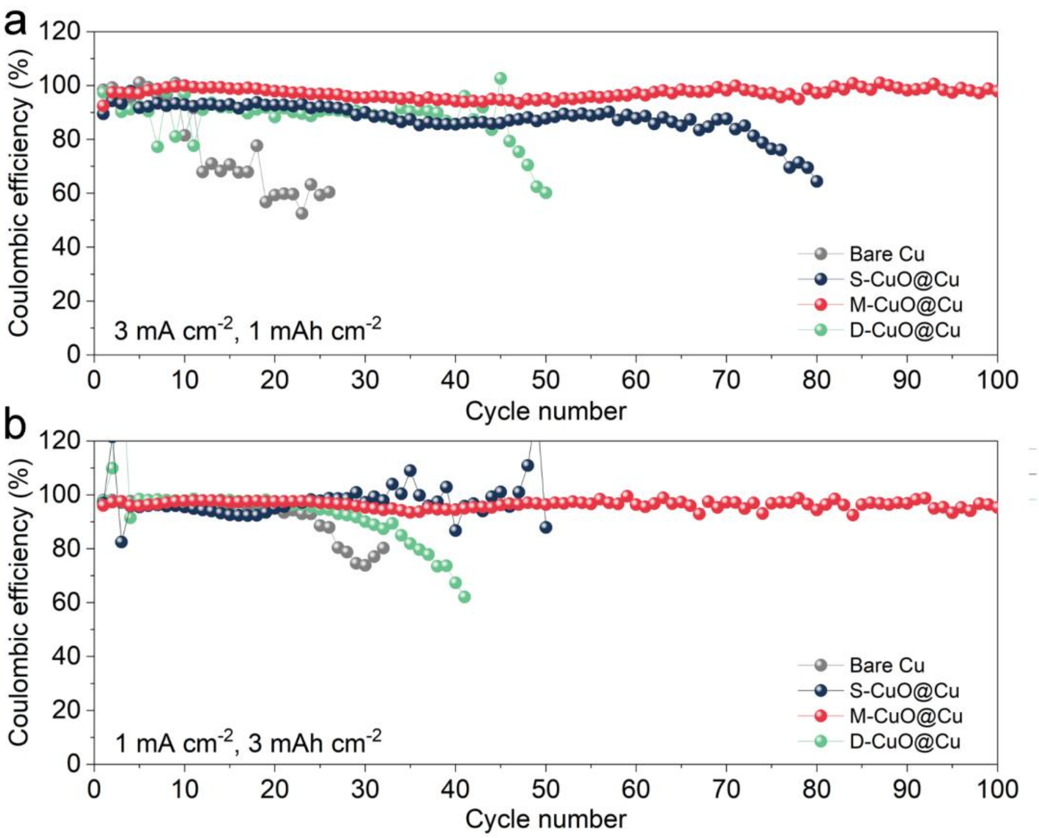

Figure S8. Comparison of CE for different electrodes at the (a) 3 mA cm^{-2} for 1 mAh cm^{-2} and (b) 1 mA cm^{-2} for 3 mAh cm^{-2}.

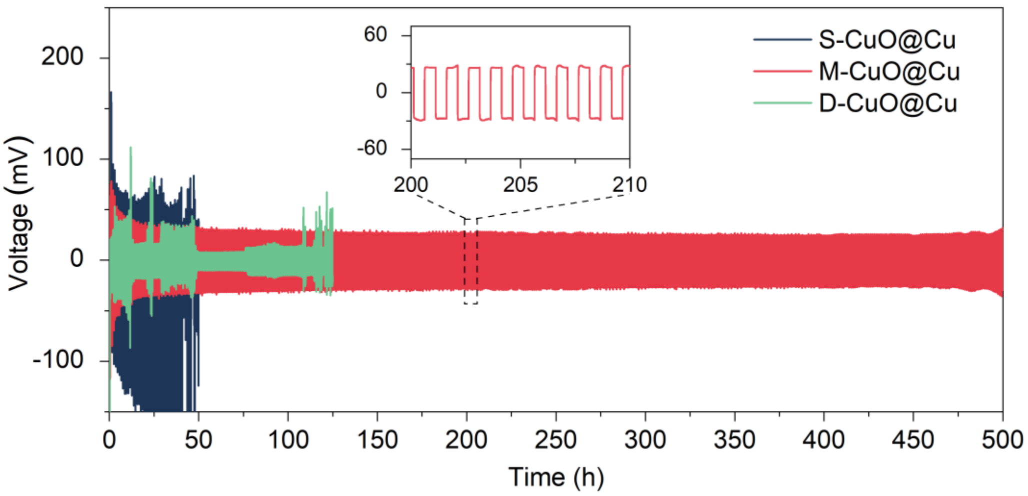

Figure S9. Cycling performance of symmetric cells based on different electrodes at 2 mA cm^{-2} for 1 mAh cm^{-2}.

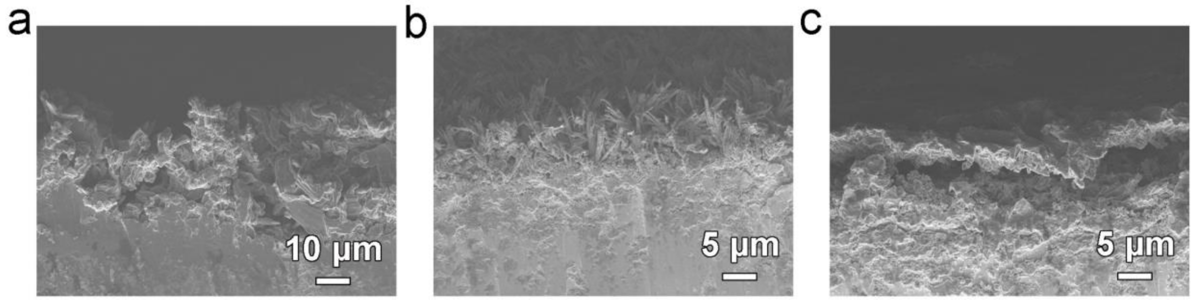

Figure S10. Cross-section SEM images of (a) S-CuO@Cu-Li, (b) M-CuO@Cu-Li, and (c) D-CuO@Cu-Li based on symmetric cells after 200 cycles at 1 mA cm^{-2} and 1 mAh cm^{-2}.

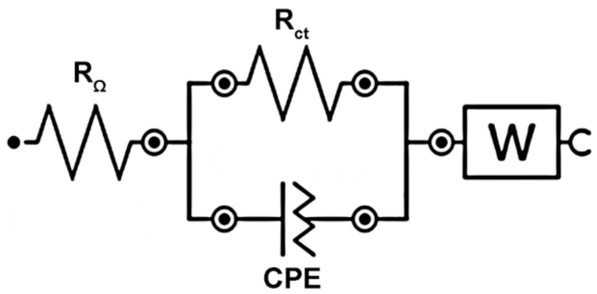

Figure S11. The equivalent circuit used for EIS analyses.

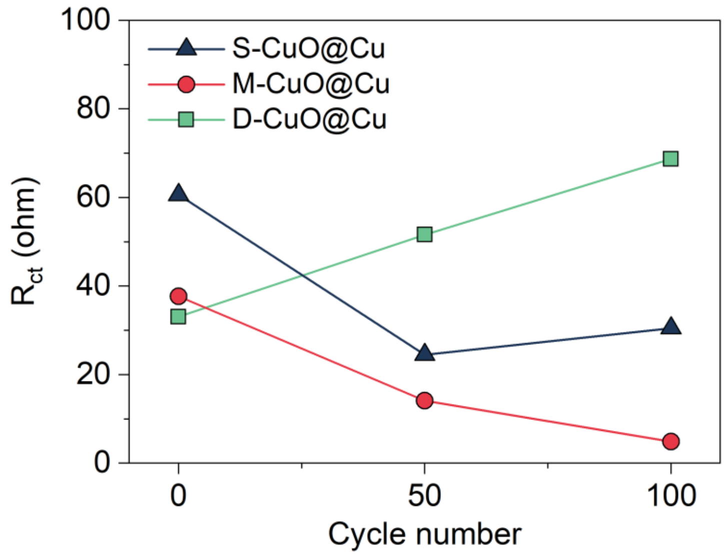

Figure S12. Change in R_{ct} of different electrodes before cycling, and after 50th and 100th cycles.

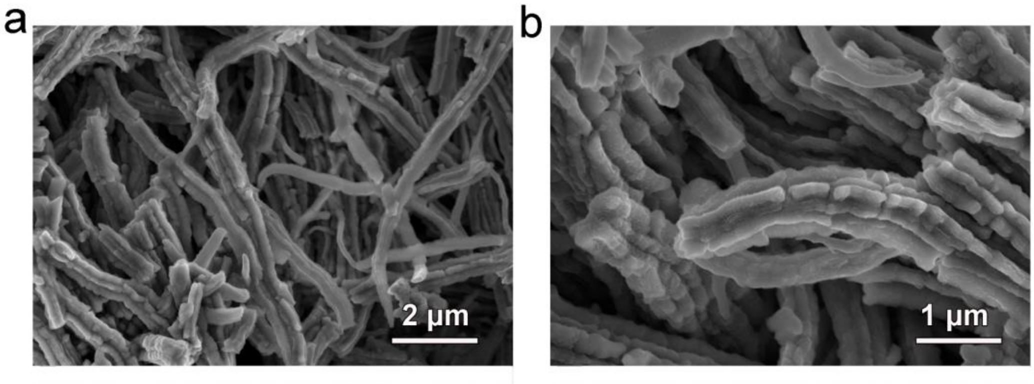

Figure S13. (a, b) SEM images of M-CuO@Cu after 0.25 mAh cm^{-2} Li plating at 0.25 mA cm^{-2}.

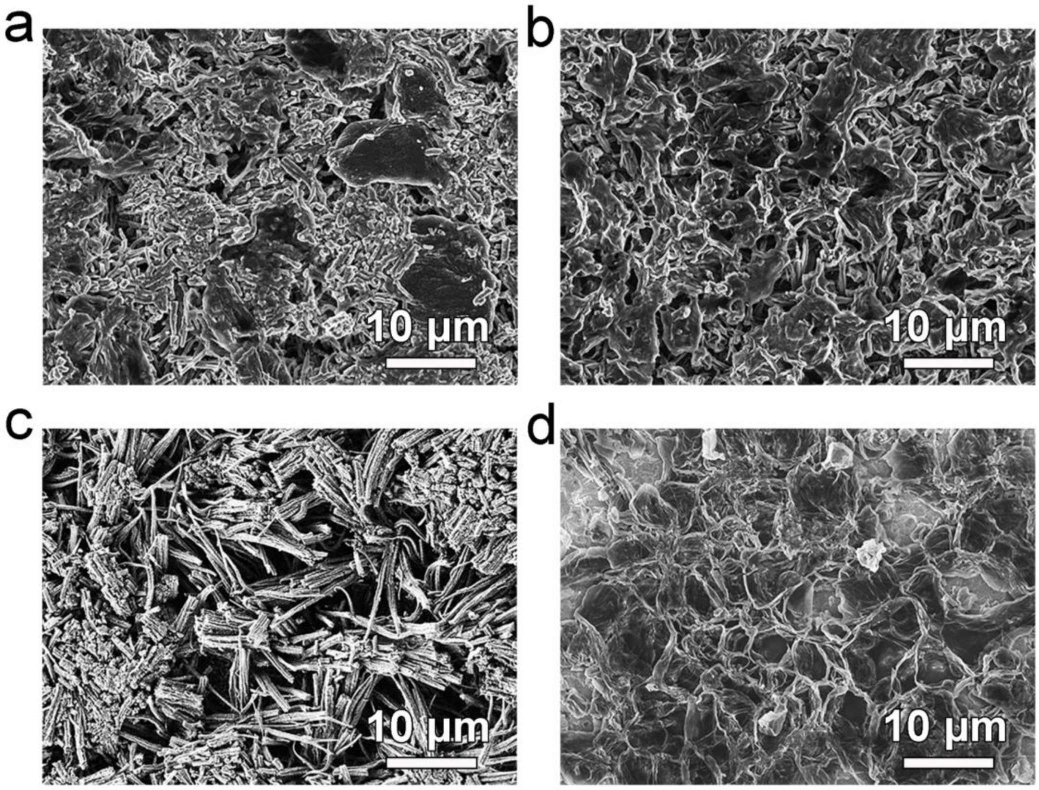

Figure S14. SEM images of (a) bare Cu, (b) S-CuO@Cu, (c) M-CuO@Cu, (d) D-CuO@Cu after 3 mAh cm^{-2} Li plating and then fully stripping at 0.25 mA cm^{-2}.

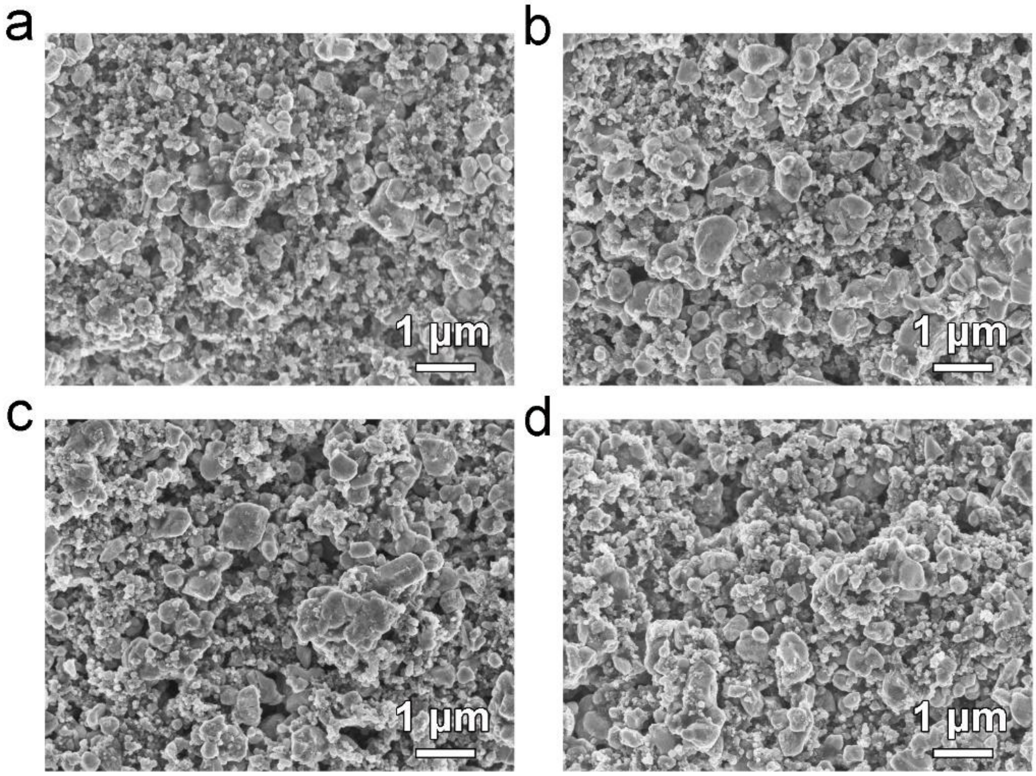

Figure S15. SEM images of the (a) pristine and (b-d) cycled LFP cathodes after 150 cycles at 1 C based on (b) LFP || S-CuO@Cu-Li, (c) LFP || M-CuO@Cu-Li, and (d) LFP || D-CuO@Cu-Li full cells.

Table S1. Comparison of cycling performance of symmetric cells based on functionalized Cu current collectors.

Current Collector	Current density (mA cm^{-2})	Areal capacity (mAh cm^{-2})	Cycling time (h)	Overpotential (mV)	Ref.
3D porous Cu current collector	1	1	400	~20	[1]
3D porous Cu current collector	1<br>2	1<br>1	800<br>535	~11<br>~15	[2]
3D porous Cu current collector	0.2	1	1000	~26	[3]
3D Cu current collector with submicron skeleton	0.2	1	600	~50	[4]
3D duplex Cu current collector	0.5<br>1	1<br>1	960<br>880	~13<br>~17	[5]
Cu-CuO-Ni current collector	0.5	0.5	580	~12	[6]
Cu foil-supported Cu_3P nanowires	1<br>2	1<br>2	1000<br>450	~30<br>~30	[7]
MOF-derived carbon framework/Ag deposited Cu current collector	0.4	0.4	500	~20	[8]
N-doped CuO nanosheet-decorated Cu current collector	1	1	600	23.1	[9]
Vertically aligned carbon nanofiber array grown on Cu current collector	1	2	500	~35	[10]
**M-CuO@Cu current collector**	**1**<br>**2**	**1**<br>**1**	**1200**<br>**500**	**10**<br>**24**	**This work**

References

[1] H. Zhao, D. Lei, Y. He, Y. Yuan, Q. Yun, B. Ni, W. Lv, B. Li, Q. Yang, F. Kang, J. Lu, *Adv. Energy Mater.* **2018**, 8, 1800266.

[2] H. Qiu, T. Tang, M. Asif, X. Huang, Y. Hou, *Adv. Funct. Mater.* **2019**, 29, 1808468.

[3] Q. Yun, Y. He, W. Lv, Y. Zhao, B. Li, F. Kang, Q. Yang, *Adv. Mater.* **2016**, 28, 6932.

[4] C. Yang, Y. Yin, S. Zhang, N. Li, Y. Guo, *Nat. Commun.* **2015**, 6, 8058.

[5] K. Lin, T. Li, S. Chiang, M. Liu, X. Qin, X. Xu, L. Zhang, F. Kang, G. Chen, B. Li, *Small* **2020**, 16, 2001784.

[6] S. Wu, Z. Zhang, M. Lan, S. Yang, J. Cheng, J. Cai, J. Shen, Y. Zhu, K. Zhang, W. Zhang, *Adv. Mater.* **2018**, 30, 1705830.

[7] P. Zhai, Y. Wei, J. Xiao, W. Liu, J. Zuo, X. Gu, W. Yang, S. Cui, B. Li, S. Yang, Y. Gong, *Adv. Energy Mater.* **2020**, 10, 1903339.

[8] J. Yun, H. Rim Shin, E. Won, H. Chol Kang, J. Lee, *Chem. Eng. J.* **2022**, 430, 132897.

[9] J. Luan, Q. Zhang, H. Yuan, D. Sun, Z. Peng, Y. Tang, X. Ji, H. Wang, *Adv. Sci.* **2019**, 6, 1901433.

[10] Y. Chen, A. Elangovan, D. Zeng, Y. Zhang, H. Ke, J. Li, Y. Sun, H. Cheng, *Adv. Funct. Mater.* **2020**, 30, 1906444.
